# Neutralization of SARS-CoV-2 by Destruction of the Prefusion Spike

**DOI:** 10.1016/j.chom.2020.06.010

**Published:** 2020-09-09

**Authors:** Jiandong Huo, Yuguang Zhao, Jingshan Ren, Daming Zhou, Helen M.E. Duyvesteyn, Helen M. Ginn, Loic Carrique, Tomas Malinauskas, Reinis R. Ruza, Pranav N.M. Shah, Tiong Kit Tan, Pramila Rijal, Naomi Coombes, Kevin R. Bewley, Julia A. Tree, Julika Radecke, Neil G. Paterson, Piyada Supasa, Juthathip Mongkolsapaya, Gavin R. Screaton, Miles Carroll, Alain Townsend, Elizabeth E. Fry, Raymond J. Owens, David I. Stuart

**Affiliations:** 1 Division of Structural Biology, University of Oxford, The Wellcome Centre for Human Genetics, Headington, Oxford, OX3 7BN, UK; 2 The Rosalind Franklin Institute, Harwell Campus, OX11 0FA, UK; 3 Protein Production UK, Research Complex at Harwell, Harwell Science & Innovation Campus, Didcot, OX11 0FA, UK; 4 MRC Human Immunology Unit, Weatherall Institute of Molecular Medicine, University of Oxford, John Radcliffe Hospital, Oxford, OX3 9DS, UK; 5 National Infection Service, Public Health England, Porton Down, Salisbury, SP4 0JG, UK; 6 Diamond Light Source Ltd, Harwell Science & Innovation Campus, Didcot, OX11 0DE, UK; 7 Nuffield Department of Medicine, Wellcome Centre for Human Genetics, University of Oxford, Oxford, OX3 7BN, UK; 8 Dengue Hemorrhagic Fever Research Unit, Office for Research and Development, Faculty of Medicine, Siriraj Hospital, Mahidol University, Bangkok 73170, Thailand; 9 Instruct-ERIC, Oxford House, Parkway Court, John Smith Drive, Oxford, OX4 2JY, UK; 10 Centre for Translational Immunology, Chinese Academy of Medical Sciences Oxford Institute, University of Oxford, Oxford OX3 7FZ, UK

**Keywords:** neutralization, antibody, SARS-CoV-2, spike, receptor binding domain, epitope, therapeutic, CR3022, cryo-electron microscopy, X-ray crystallography

## Abstract

There are as yet no licensed therapeutics for the COVID-19 pandemic. The causal coronavirus (SARS-CoV-2) binds host cells via a trimeric spike whose receptor binding domain (RBD) recognizes angiotensin-converting enzyme 2, initiating conformational changes that drive membrane fusion. We find that the monoclonal antibody CR3022 binds the RBD tightly, neutralizing SARS-CoV-2, and report the crystal structure at 2.4 Å of the Fab/RBD complex. Some crystals are suitable for screening for entry-blocking inhibitors. The highly conserved, structure-stabilizing CR3022 epitope is inaccessible in the prefusion spike, suggesting that CR3022 binding facilitates conversion to the fusion-incompetent post-fusion state. Cryogenic electron microscopy (cryo-EM) analysis confirms that incubation of spike with CR3022 Fab leads to destruction of the prefusion trimer. Presentation of this cryptic epitope in an RBD-based vaccine might advantageously focus immune responses. Binders at this epitope could be useful therapeutically, possibly in synergy with an antibody that blocks receptor attachment.

## Highlights

• CR3022 binds the RBD of SARS-CoV-2 and shows strong neutralization
• Neutralization is by destroying the prefusion spike conformation
• CR3022 binds a highly conserved epitope that is inaccessible in prefusion spike protein
• CR3022 could have therapeutic potential alone or in synergy with a receptor blocker

## Graphical Abstract

**Figure:**
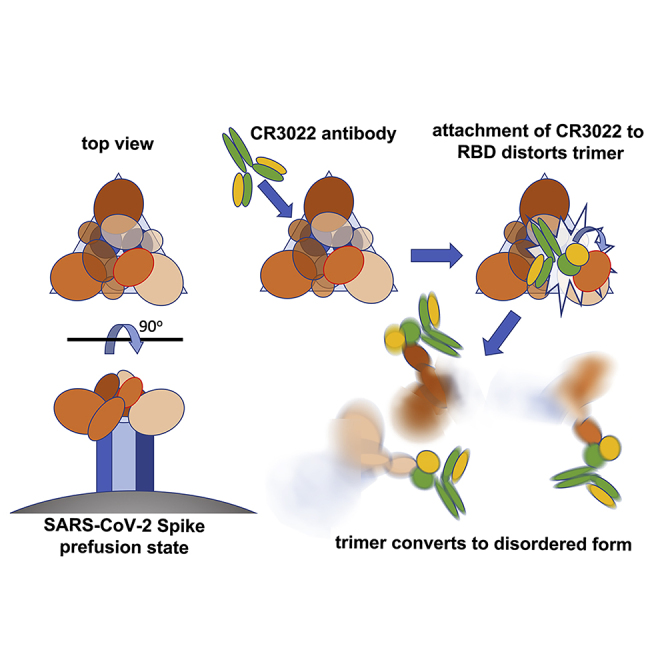

## Teaser

Huo et al. find that the antibody CR3022 binds tightly to the receptor binding domain of the SARS-CoV-2 spike at a site different to that used by the receptor. CR3022 effectively neutralizes the virus, and cryo-EM reveals that it disrupts the spike. Such antibodies could have potential as COVID-19 therapeutics.

## Introduction

**Figure 1:**
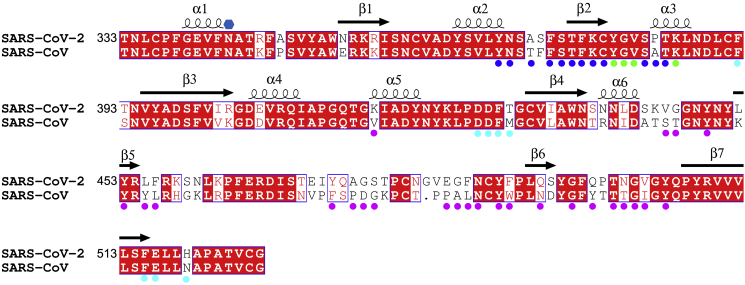
Sequence Alignment between the RBDs of SARS-CoV(-1) and SARS-CoV-2 Residue numbers are those of SARS-CoV-2 RBD. Conserved amino acids have a red background, secondary structures are labeled on the top of the sequence, and the glycosylation site is marked with a blue hexagon. Residues involved in receptor binding are marked with magenta disks. Blue disks mark the residues involved in interactions with the CR3022 heavy chain (Vh), cyan disks mark the residues interacting with the CR3022 light chain (Vl), and green disks mark those with both chains.

Incursion of animal (usually bat)-derived coronaviruses into the human population has caused several outbreaks of severe disease, starting with severe acute respiratory syndrome (SARS) in 2002 (Menachery et al., 2015). In late 2019, a highly infectious illness with cold-like symptoms progressing to pneumonia and acute respiratory failure, resulting in an estimated 6% overall death rate (Baud et al., 2020), with higher mortality among the elderly and immunocompromised populations, was identified and confirmed as a pandemic by the WHO on March 11, 2020. The etiological agent is a novel coronavirus (SARS-CoV-2) belonging to lineage B betacoronaviruses and sharing 88% sequence identity with bat coronaviruses (Lu et al., 2020a). The heavily glycosylated trimeric surface spike protein mediates viral entry into the host cell. It is a large type I transmembrane glycoprotein (the ectodomain alone comprises over 1,200 residues) (Wrapp et al., 2020). It is made as a single polypeptide and then cleaved by host proteases to yield an N-terminal S1 region and the C-terminal S2 region. Spike exists initially in a pre-fusion state where the domains of S1 cloak the upper portion of the spike with the relatively small (~22 kDa) S1 receptor binding domain (RBD) nestled at the tip. The RBD is predominantly in a “down” state where the receptor binding site is inaccessible; however, it appears that it stochastically flips up with a hinge-like motion transiently presenting the angiotensin-converting enzyme 2 (ACE2) receptor binding site (Roy, 2020; Song et al., 2018; Walls et al., 2020; Wrapp et al., 2020). ACE2 acts as a functional receptor for both SARS-CoV-1 and SARS-CoV-2, binding to the latter with a 10- to 20-fold higher affinity (K_D_ of ~15 nM), possibly contributing to its ease of transmission (Song et al., 2018; Wrapp et al., 2020). There is 73% sequence identity between the RBDs of SARS-CoV-1 and SARS-CoV-2 (Figure 1). When ACE2 locks on, it holds the RBD “up,” destabilizing the S1 cloak and possibly favoring conversion to a post-fusion form where the S2 subunit, through massive conformational changes, propels its fusion domain upward to engage with the host membrane, casting off S1 in the process (Song et al., 2018; Wrapp et al., 2020). Structural studies of the RBD in complex with ACE2 (Lan et al., 2020; Wang et al., 2020; Yan et al., 2020) show that it is recognized by the extracellular peptidase domain (PD) of ACE2 through mainly polar interactions. The spike protein is an attractive candidate for both vaccine development and immunotherapy. Potent nanomolar affinity-neutralizing human monoclonal antibodies against the SARS-CoV-1 RBD have been identified that attach at the ACE2 receptor binding site (including M396, CR3014, and 80R [ter Meulen et al., 2006; Sui et al., 2004; Zhu et al., 2007]). For example, 80R binds with nanomolar affinity, prevents binding to ACE2 and the formation of syncytia *in vitro*, and inhibits viral replication *in vivo* (Sui et al., 2004). However, despite the two viruses sharing the same ACE2 receptor, these ACE2-blocking antibodies do not bind SARS-CoV-2 RBDs (Wrapp et al., 2020). In contrast, CR3022, a SARS-CoV-1-specific monoclonal selected from a single-chain Fv phage display library constructed from lymphocytes of a convalescent SARS patient and reconstructed into IgG1 format (ter Meulen et al., 2006), has been reported to cross-react strongly, binding to the RBD of SARS-CoV-2 with a K_D_ of 6.3 nM (Tian et al., 2020) while not competing with the binding of ACE2 (ter Meulen et al., 2006). Furthermore, although SARS-CoV-1 escape mutations could be readily generated for ACE2-blocking CR3014, no escape mutations could be generated for CR3022, preventing mapping of its epitope (ter Meulen et al., 2006). Furthermore, a natural mutation of SARS-CoV-2 has now been detected at residue 495 (Y→N) (GISAID [Shu and McCauley, 2017]: Accession ID: EPI_ISL_429783), which forms part of the ACE2 binding epitope. Finally, CR3022 and CR3014 act synergistically to neutralize SARS-CoV-1 with extreme potency (ter Meulen et al., 2006). While this work was being prepared for publication, a paper was published reporting that CR3022 does not neutralize SARS-CoV-2 and describing the structure of the complex with the RBD at 3.1 Å resolution (Yuan et al., 2020). Here, we report crystallographic analysis to significantly higher resolution, use a different neutralization assay to show that CR3022 does neutralize SARS-CoV-2, and use cryogenic electron microscopy (cryo-EM) analysis of the interaction of CR3022 with the full spike ectodomain to demonstrate a mechanism of neutralization not seen before for coronaviruses. Taken together, these observations suggest that the CR3022 epitope should be a major target for therapeutic antibodies.

## Results

### CR3022 Binds Tightly to the RBD and Allosterically Perturbs ACE2 Binding

To understand how CR3022 works, we first investigated the interaction of CR3022 Fab with isolated recombinant SARS-CoV-2 RBD, both alone and in the presence of ACE2. Surface plasmon resonance (SPR) measurements (Figure S1; STAR Methods) confirmed that CR3022 binding to the RBD is strong (although weaker than the binding reported to SARS-CoV-1 [ter Meulen et al., 2006]), with a slight variation according to whether CR3022 or the RBD is used as the analyte (K_D_ = 30 nM and 15 nM, respectively, derived from the kinetic data in Table S1). An independent measure using Bio-Layer Interferometry (BLI) with the RBD as analyte gave a K_D_ of 19 nM (Figure S1; STAR Methods). These values are quite similar to those reported by Tian et al. (2020) (6.6 nM), whereas weaker binding (K_D_ ~115 nM) was reported recently by Yuan et al. (2020). The use of SPR to perform a competition assay revealed that the binding of ACE2 to the RBD is perturbed by the presence of CR3022 (Figure S1). The presence of ACE2 slows the binding of CR3022 to the RBD and accelerates the dissociation. Similarly, the release of ACE2 from the RBD is accelerated by the presence of CR3022. These observations are suggestive of an allosteric effect between ACE2 and CR3022.

### CR3022 Neutralizes SARS-CoV-2

**Figure 2:**
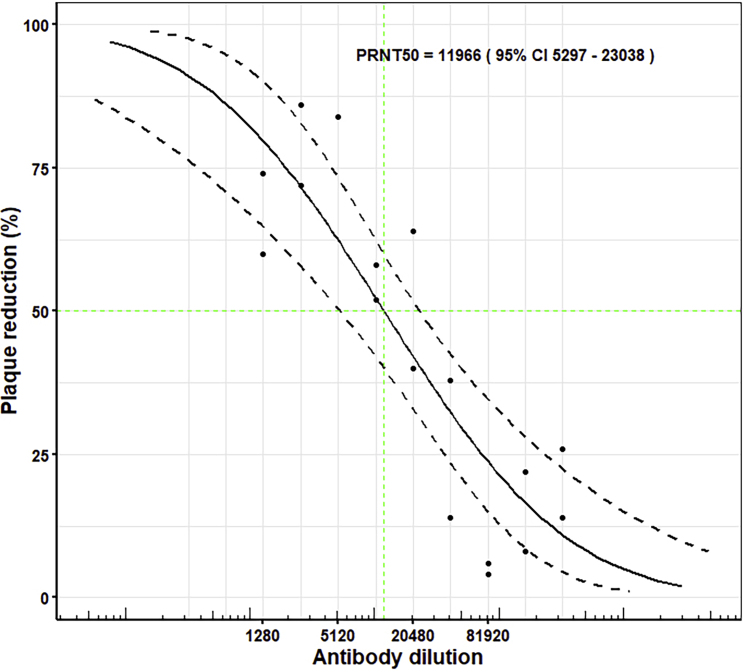
Dose-Response Curve for PRNT with CR3022 For CR3022 at a starting concentration of 1.36mg/mL, the dilutions used were from 1:160 to 1:327,680. The probit mid-point is 1:11,966 (95% confidence intervals: 5,297–23,038).

A plaque-reduction neutralization test (PRNT) using SARS-CoV-2 virus and CR3022 showed a probit mid-point PRNT_50_ of 1:11,966 (95% confidence interval 5,297–23,038) for a starting concentration of 1.36 mg/mL (calculated according to Grist [1966]), superior to that of the NIBSC international standard positive control used by Public Health England (MERS convalescent serum that gives a PRNT_50_ of 1:874 [95% confidence interval 663–1,220]; Figure 2; Table S2; STAR Methods). This corresponds to 50% neutralization at ~0.114 μg/mL (~1 nM) exceeding the 11 μg/mL reported by ter Meulen et al. (2006) for SARS-CoV-1; however, as discussed below, it is in apparent disagreement with the result reported recently by Yuan et al. (2020). In light of this discrepancy, further neutralization tests were performed to rule out differences in the assay with regard to antibody/virus contact time. Repeated PRNT tests deliberately using three different batches of CR3022 gave similar results (Table S2), and leaving the virus/antibody mix in place throughout the incubation on the plate and removing the antibody after 1 h also gave similar results (PRNT_50_ values of 1:4,666 and 1:6,504, respectively; see STAR Methods for experimental details; Figure S2). In summary, all of these results, taking into consideration the different CR3022 starting concentrations, were within the same confidence levels. Following these experiments, a commercial source of antibody CR3022 (Creative Biolabs) was tested (using the same method and on the same date as the above wash and leave' experiment, with a starting concentration of 1 mg/mL). This gave markedly weaker neutralization: PRNT_50_ 1:27 leaving the antibody on the plate and 1:285 washing it off. Note that in both cases the neutralization was slightly higher when the antibody was washed off. Although the differences were within the confidence levels of the experiments, it is possible that this reflects unbound virus remaining in the inoculum being washed off.

### Structure Determination of the RBD-CR3022 Fab Complex

We determined the crystal structure of the SARS-CoV-2 spike RBD-CR3022 Fab complex (Table S3; STAR Methods) to investigate the relationship between the binding epitopes of ACE2 and CR3022. Crystals grew rapidly and consistently. Two crystal forms grew in the same drop. The solvent content of the crystal form solved first was unusually high (ca 87%), with the ACE2 binding site exposed to large continuous solvent channels within the crystal lattice (Figure S3). These crystals therefore offer a promising vehicle for crystallographic screening to identify potential therapeutics that could act to block virus attachment. The current analysis of this crystal form is at 4.4 Å resolution, and so, to avoid overfitting, refinement used a real-space refinement algorithm to optimize the phases (Vagabond, HMG unpublished; STAR Methods). This, together with the favorable observation to parameter ratio resulting from the exceptionally high solvent content, meant that the map was of very high quality, allowing reliable structural interpretation (Figure S4; STAR Methods). Full interpretation of the detailed interactions between CR3022 and the RBD was enabled by the second crystal form which diffracted to high resolution, 2.4 Å, and the structure of which was refined to give an R-work/R-free of 0.213/0.239 and good stereochemistry (Figure S4; Table S3; STAR Methods). The structure is similar to that reported by Yuan et al., (2020); the RMSD in Cαs for the RBD is 0.5 Å, whereas for the CR3022 heavy chain it is 1.1 Å and for the light chain 0.7 Å. There are also some differences in the overall interaction in comparison with that structure; after overlapping the RBD, the angular differences for the variable domains are 5.5° and 8°.

### CR3022 Binding Epitope Is Highly Conserved and Inaccessible in Prefusion S Protein

**Figure 3:**
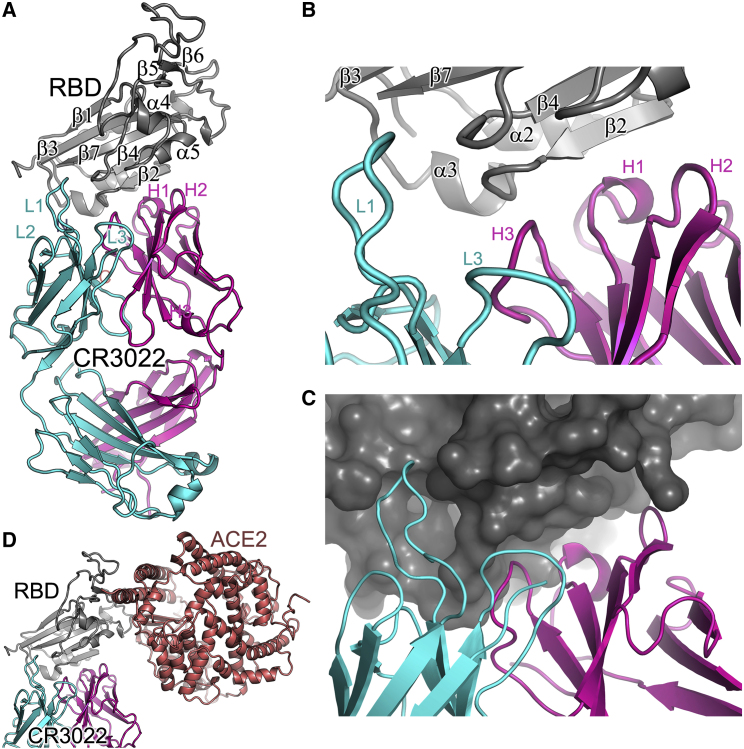
Overall Structure of RBD/CR3022 Complex (A) Ribbon diagram showing the structure of the RBD/CR3022 complex with the RBD shown in gray, CR3022 heavy chain in magenta, and light chain in cyan. The heavy chain CDR1-3 are labeled as H1–H3 and the light chain CDR1-3 as L1–L3 (where visible). (B) Closeup of the antigen-antibody binding interface in cartoon representation. (C) Similar view to (B), but showing the RBD as a surface. (D) The RBD of the RBD/ACE2 complex has been overlapped with the RBD of the RBD/CR3022 complex to show the relative positions of the antigenic and receptor binding sites. ACE2 is drawn as a salmon ribbon.

**Figure 4:**
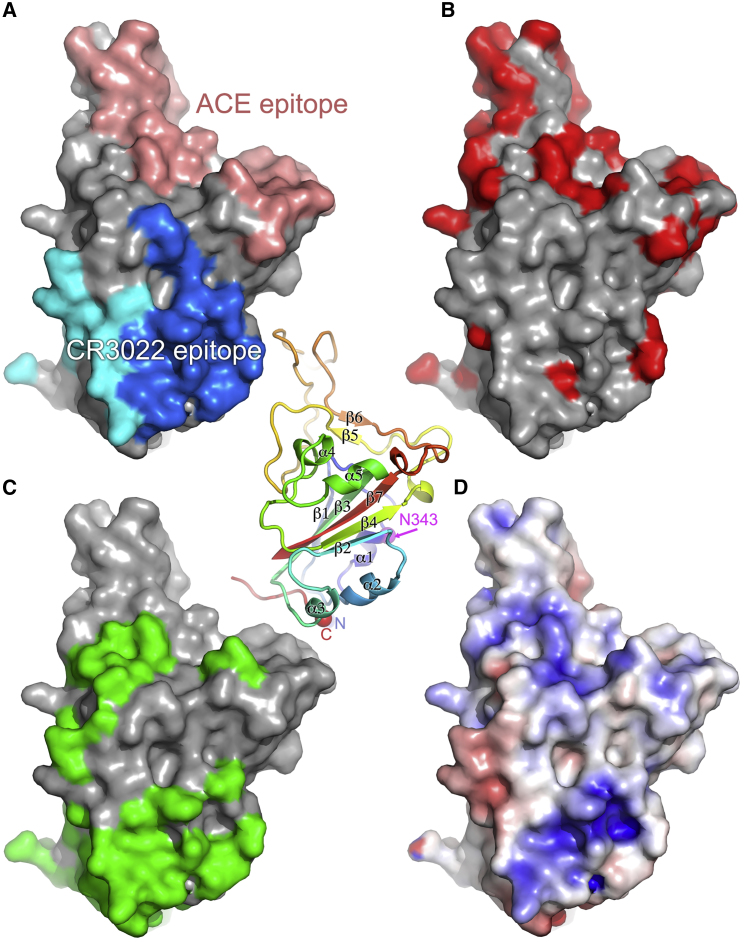
Surface Properties of SARS-CoV-2 RBD The central panel is a cartoon depiction rainbow colored from blue for the N terminus to red for the C terminus; the view is the same as for (A)–(D). The secondary structure is labeled along with the glycosylated residue N343 (in magenta) and the position of the domain termini (N and C). (A) Surface representation of RBD, with the solvent-accessible area buried by ACE2 receptor binding colored in salmon and that buried by CR3022 (heavy chain in blue and light chain in cyan). (B) Sequence differences shown in red between SARS-CoV and SARS-CoV-2 RBDs, mapped on the surface of SARS-CoV-2 RBD. (C) The surface buried in the pre-fusion conformation of the spike shown in green. (D) The electrostatic surface of SARS-CoV-2 RBD contoured at ± 5 T/e (red, negative; blue, positive).

**Figure 5:**
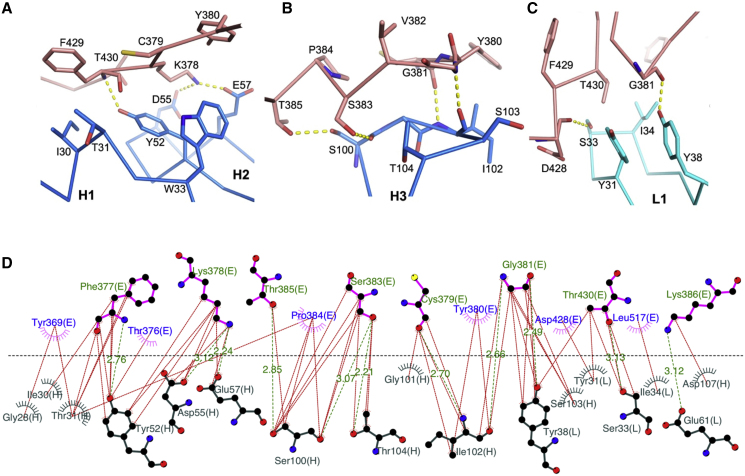
Details of Contacts between the RBD and CR3022 (A and B) Contacts of the RBD with CR3022 heavy chain CDR1 (H1) and CDR2 (H2) (A), and with CDR3 (H3) (B). (C) Interactions between the RBD and the light chain CDR1 (L1). Main chain backbones are shown as thinner sticks and side chains as thick sticks (RBD, salmon; heavy chain, blue; light chain, cyan). The yellow broken sticks represent hydrogen bonds or salt bridges. (D) Ligplot (Laskowski and Swindells, 2011) representation of the interface details (chain identifiers: L, CR3022 light chain; H, CR3022 heavy chain; E, RBD).

The high-resolution structure is shown in Figure 3. There are two complexes in the crystal asymmetric unit with residues 331–529 in one RBD, 332–445, and 448–532 in the other RBD well defined, whereas residues 133–136 of the CR3022 heavy chains are disordered. The RBD has a very similar structure to that seen in the complex of SARS-CoV-2 RBD with ACE2, RMSD for 194 Cα atoms of 0.6 Å (PDB: 6M0J [Lan et al., 2020]), and an RMSD of 1.1 Å compared to the SARS CoV-1 RBD (PDB: 2AJF [Li et al., 2005]). Only minor conformational changes are introduced by binding to CR3022, at residues 381–390. The RBD was deglycosylated (STAR Methods) to leave a single saccharide unit at each of the N-linked glycosylation sites clearly seen at N331 and N343 (Figure S4). CR3022 attaches to the RBD surface orthogonal to the ACE2 receptor binding site. There is no overlap between the epitopes, and indeed both the Fab and ACE2 ectodomain can bind without clashing (Figure 3D) (Tian et al., 2020). Such independence of the ACE2 binding site has been reported recently for another SARS-CoV-2 neutralizing antibody, 47D11 (Okba et al., 2020). The Fab complex interface buries 990 Å^2^ of surface area (600 and 390 Å^2^ by the heavy and light chains, respectively; Figures 4 and S5), somewhat more than the RBD-ACE2 interface, which covers 850 Å^2^ (PDB: 6M0J [Lan et al., 2020]). Typical of a Fab complex, the interaction is mediated by the antibody CDR loops, which fit well into the rather sculpted surface of the RBD (Figures 3B and 3C). The heavy chain CDR1, 2, and 3 make contacts to residues from α2, β2, and α3 (residues 369–386), whereas two of the light-chain CDRs (1 and 2) interact mainly with residues from the β2-α3 loop, α3 (380-392), and the α5-β4 loop (427–430) (Figures 1, 3, and 5). A total of 16 residues from the heavy chain and 14 from the light chain cement the interaction, with 26 residues from the RBD. For the heavy chain these potentially form seven H-bonds and three salt bridges, the latter from D55 and E57 (CDR2) to K378 of the RBD. The light-chain interface comprises six H-bonds and a single salt bridge between E61 (CDR2) and K386 of the RBD. The binding is consolidated by a number of hydrophobic interactions (Figure 5). There are slight differences in the interactions between these and those reported by Yuan et al. (2020); for instance, the contact area for the light chain-RBD differs by ~12.5% between the two structures. Of the 26 residues involved in the interaction, 23 are conserved between SARS-CoV-1 and SARS-CoV-2 (Figures 1 and 4). The CR3022 epitope is much more conserved than that of the receptor-blocking anti-SARS-CoV-1 antibody 80R, for which only 13 of the 29 interacting residues are conserved (Hwang et al., 2006), in-line with the lack of cross reactivity observed for the latter.

**Figure 6:**
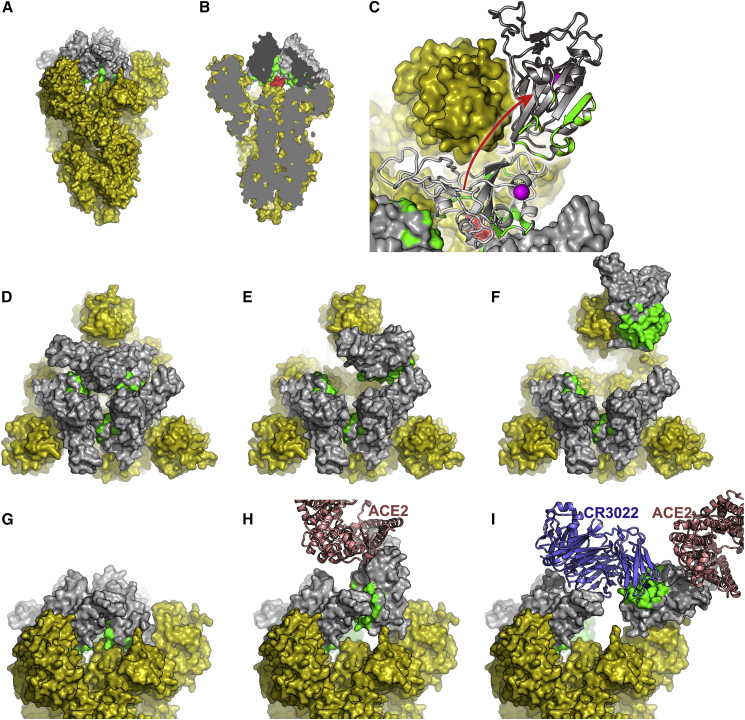
The CR3022 Binding Regions Are Inaccessible in the Pre-fusion Form of the S Protein (A)–(C) An overview. (A) The pre-fusion state of the S protein with all RBDs in the down conformation (generated by superposing our RBD structure on the pre-fusion trimer [Wrapp et al., 2020]). The viral membrane would be at the bottom of the picture. All of S1 and S2 are shown in yellow apart from the RBD, which is shown in gray, with the CR3022 epitope colored green. (B) A cut-way of the trimer showing, in red and the di-peptide (residues 986–987), which has been mutated to PP to inhibit conversion to the post-fusion state. Note the proximity to the CR3022 epitope. (C) A top view of the molecule (also used for [D]–[F]). One of the RBDs has been drawn in light gray in the down configuration and hinged up in dark gray, using the motion about the hinge axis observed for several coronavirus spikes, but extending the motion sufficiently to allow CR3022 to bind. The PP motif is shown in red and the glycosylated residue N343 in magenta. (D–F) The trimer viewed from above. All RBDs down (D), one RBD up (E), and one RBD rotated (F) (as in [C]) to allow access to CR3022. (G–I) Equivalent structures to (D)–(F) but viewed from the side, in (H) bound ACE2 is shown and in (I) CR3022 is shown.

The reason for the conservation of the CR3022 epitope becomes clear in the context of the complete pre-fusion S structure (PDB IDs: 6VSB [Wrapp et al., 2020], 6VXX, 6VYB [Walls et al., 2020]) where the epitope is inaccessible (Figure 6). When the RBD is in the down configuration, the CR3022 epitope is packed tightly against another RBD of the trimer and the N-terminal domain (NTD) of the neighboring protomer. In the structure of the pre-fusion form of trimeric spike, the majority of RBDs are down, although presumably stochastically one could be up (Walls et al., 2020; Wrapp et al., 2020). The structure of a SARS-CoV-1 complex with ACE2 ectodomain shows that this up configuration is competent to bind receptor and that there is a family of up orientations with significantly different hinge angles (Song et al., 2018). However, the CR3022 epitope remains largely inaccessible even in the up configuration. Modeling the rotation of the RBD required to enable Fab interaction in the context of the spike trimer showed a rotation corresponding to a >60° further declination from the central vertical axis was required, beyond that observed previously (Walls et al., 2020; Wrapp et al., 2020) (Figure 6I), although this might be partly mitigated by more complex movements of the RBD, and if more than one RBD is in the up configuration then this requirement would be relaxed somewhat. Because locking the up state by receptor-blocking antibodies is thought to destabilize the pre-fusion state (Walls et al., 2019) binding of CR3022 presumably introduces further destabilization, leading to a premature conversion to the post-fusion state, inactivating the virus. CR3022 and ACE2-blocking antibodies can bind independently but both induce an up conformation, presumably explaining the observed synergy between binding at the two sites (ter Meulen et al., 2006).

### Mechanism of Neutralization of SARS-CoV-2 by CR3022 Confirmed by Cryo-EM

**Figure 7:**
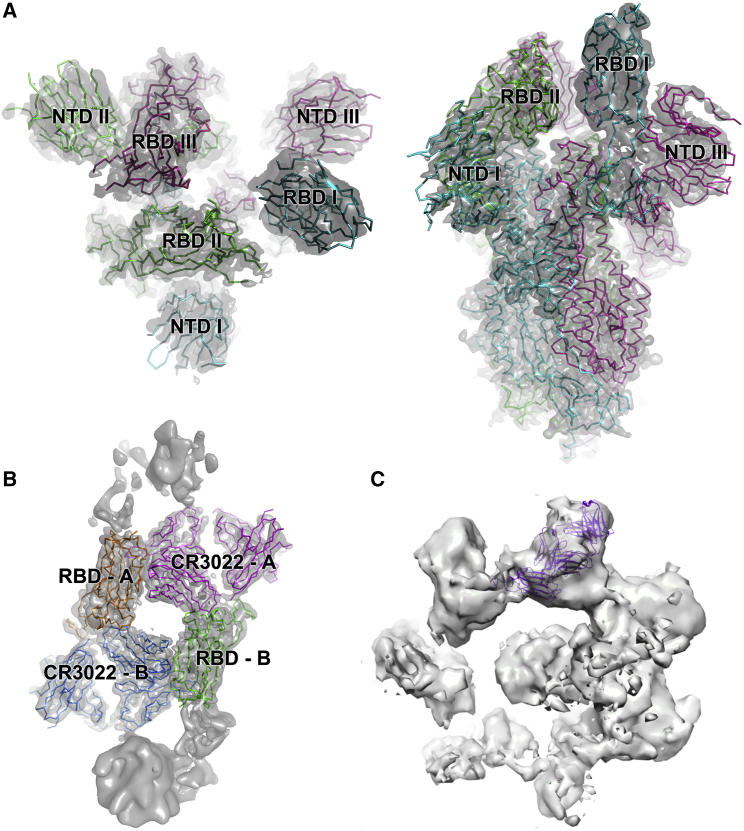
Cryo-EM Reconstructions (A) and (B) are derived from the 50 min incubation, (C) from the 3 h incubation. (A) Cryo-EM map and fitted model of the prefusion spike: left top-view, right side-view. Note RBD I is in the up conformation. (B) Cryo-EM map and fitted model of the dimeric RBD/CR3022 complex, with each monomer labeled A and B. (C) Reconstruction from 3-h incubation dataset to indicate how the CR3022 Fab/RBD complex might be accommodated within one oligomeric unit.

To test if CR3022 binding destabilizes the prefusion state of spike, the ectodomain construct described previously (Wrapp et al., 2020) was used to produce glycosylated protein in HEK cells (STAR Methods). Cryo-EM screening showed that the protein was in the trimeric prefusion conformation. Spike was then mixed with an excess of CR3022 Fab and incubated at room temperature, with aliquots being taken at 50 min and 3 h. Aliquots were immediately applied to cryo-EM grids and frozen (STAR Methods). For the 50 min incubation, collection of a substantial amount of data allowed unbiased particle picking and 2D classification that revealed two major structural classes with a similar number in each: (1) the prefusion conformation and (2) a radically different structure (Figure S6; Table S4; STAR Methods). Detailed analysis of the prefusion conformation led to a structure at a nominal resolution of 3.4 Å (FSC = 0.143), based on a broad distribution of orientations, that revealed the same predominant RBD pattern (one up and two down) previously seen (Wrapp et al., 2020) with no evidence of CR3022 binding (Figure 7A). Analysis of the other major particle class revealed strong preferential orientation of the particles on the grid (Figure S6C). Despite this, a reconstruction with a nominal resolution of 3.9 Å within the plane of the grid, and perhaps 7 Å resolution in the perpendicular direction (Figure S6G), could be produced, which allowed the unambiguous fitting of the CR3022-RBD complex (Figure 7B). Note that in addition there is less well-defined density attached to the RBD in a suitable position to correspond to the spike N-terminal domain (Wrapp et al., 2020). These structures are no longer trimeric; rather, two complexes associate to form an approximately symmetric dimer (however, application of C2 symmetry in the reconstruction process did not noticeably improve the resolution). The interactions responsible for dimerization involve the ACE2 binding site on the RBD and the elbow of the Fab; however, the interaction does not occur in our low-resolution crystal form and is therefore probably extremely weak and not biologically significant. Because conversion to the post-fusion conformation leads to dissociation of S1 (which includes the N-terminal domain and RBD), these results confirm that CR3022 destabilizes the prefusion spike conformation. Further evidence of this is provided by analysis of data collected after 3 h of incubation. By this point there were no intact trimers remaining, and a heterogeneous range of oligomeric assemblies had appeared, which we were not able to interpret in detail but are consistent with the lateral assembly of Fab/RBD complexes (Figure S7). Note that the relatively slow kinetics will not be representative of events *in vivo*, where the conversion might be accelerated by the elevated temperature and the absence of the mutations that were added to this construct to stabilize the prefusion state (Kirchdoerfer et al., 2018; Pallesen et al., 2017; Wrapp et al., 2020).

## Discussion

Until now, the only documented mechanism of neutralization of coronaviruses has been through blocking receptor attachment. In the case of SARS-CoV-1, this is achieved by presentation of the RBD of the spike in an up conformation. Although not yet confirmed for SARS-CoV-2, it is very likely that a similar mechanism can apply. Here we define a second class of neutralizers that bind a highly conserved epitope (Figure 1) and can therefore act against both SARS-CoV-1 and SARS-CoV-2 (CR3022 was first identified as a neutralizing antibody against SARS-CoV-1 [ter Meulen et al., 2006]). We find that binding of CR3022 to the isolated RBD is tight (~20 nM), and the crystal structure of the complex reveals the atomic details of the interactions. Despite the spatial separation of the CR3022 and ACE2 epitopes, we find an allosteric effect between the two binding events. The role of the CR3022 epitope in stabilizing the prefusion spike trimer explains why it has, to date, proved impossible to generate mutations that escape binding of the antibody (ter Meulen et al., 2006).

Although in our assay CR3022 strongly neutralizes SARS-CoV-2, a recent paper (Yuan et al., 2020) reported an alternative assay that did not detect neutralization. We tested whether the removal of the antibody/virus mix after adsorption to the indicator cells, performed by Yuan et al., before incubating to allow cytopathic effect (CPE) to develop, would explain this difference. This would be in line with the distinction previously seen between neutralization tests for influenza virus by antibodies that bind the stem of hemagglutinin and therefore do not block receptor binding (Thomson et al., 2012). These antibodies did not appear to be neutralizing when tested with the standard WHO neutralization assay, in which similar to Yuan et al., the inoculum of virus/antibody is washed out before development of CPE. Neutralization was observed, however, when the antibodies were left in the assay during incubation to produce CPE. Performing side-by-side PRNT experiments, leaving the antibody/virus mix in place, and washing it off did not, however, show a significant difference. In fact, the neutralization was marginally stronger when excess antibody and virus was washed off. To check if there were issues related to the reproducibility, we performed neutralization tests on three separate batches of CR3022. All gave essentially indistinguishable results (Table S2); however, when we tested commercially sourced CR3022 (Creative BioLabs, USA; CAT#: MRO-1214LC), the neutralization was markedly reduced, perhaps due to improperly folded antibody. It is possible the loss of neutralization ability with commercial antibody could be related to the report that CR3022 does not neutralize SARS-CoV-2 (Yuan et al., 2020). In addition, we note that in all the PRNT tests performed, CR3022 appears to give strong but incomplete (90% plaque reduction) neutralization. Such partial neutralization has been reported before, for antibodies against Ebola virus, which nonetheless confer profound protection (Rijal et al., 2019; Saphire et al., 2018). Given the mechanism of neutralization we rationalize that this arises from the kinetic limitation of antibody binding and spike destruction, as seen by cryo-EM, where in the absence of ACE2, the CR3022 Fab destroys the prefusion-stabilized trimer with T_1/2_ ~1 h at room temperature. This might also lead to slightly higher neutralization when antibody and (non-inactivated) virus is washed off the cells after 1 h. In summary CR3022 neutralizes SARS-CoV-2, but via an unusual mechanism which some assays appear to detect poorly, as observed by Yuan et al., 2020. It is now important to establish how effective this mechanism is at controlling viral infection.

With monoclonal antibodies now recognized as potential antivirals (Lu et al., 2020b; Qiu et al., 2014; Salazar et al., 2017), our results suggest that CR3022 could be of immediate utility because the mechanism of neutralization will be unusually resistant to virus escape. In contrast, antibodies which compete with ACE2 (whose epitope on SARS-CoV-2 is reported to have already shown mutation at residue 495 [GISAID: Accession ID: EPI_ISL_429783 (Shu and McCauley, 2017)]), are likely to be susceptible to escape. Furthermore, with knowledge of the detailed structure of the epitope presented here, a higher affinity version of CR3022 might be engineered. Alternatively, because the same mechanism of neutralization is likely to be used by other antibodies, a more potent monoclonal antibody targeting the same epitope might be found (for instance by screening for competition with CR3022). Additionally, because this epitope is sterically and functionally independent of the well-established receptor-blocking neutralizing antibody epitope, there is considerable scope for therapeutic synergy between antibodies targeting the two epitopes (indeed, this type of synergy has been described for SARS-CoV-1 [ter Meulen et al., 2006]). Moreover, it has been reported (Wan et al., 2020) that antibody-mediated enhancement occurs via antibodies that mimic receptor attachment whereas CR3022-like binding might circumvent this by pre-attachment conversion to the post-fusion state. Finally, display of this epitope on an RBD-based vaccine antigen might focus immune responses, conceivably mitigating the immunopathology reported for SARS-CoV-1 (Perlman and Dandekar, 2005; Tseng et al., 2012).

## STAR★Methods

### Key Resources Table

**Table:** 

REAGENT or RESOURCE	SOURCE	IDENTIFIER
**Antibodies**		

Anti-SARS-CoV Antibody Fab Fragment (CR3022)	Creative BioLabs, USA	Cat#: MRO-1214LC
CR3022 Fab	This paper	N/A
CR3022 IgG	This paper	N/A
E08R Fab	This paper	N/A

**Bacterial and Virus Strains**		

SARS-CoV-2 (Australia/VIC01/2020)	Caly et al., 2020	N/A
DH5α bacteria	Thermo Fisher	Cat# 18263012

**Biological Samples**		

His-tagged RBD	This paper	N/A
Biotinylated RBD	This paper	N/A
His-tagged ACE2	This paper	N/A
ACE2-hIgG1Fc	This paper	N/A

**Chemicals, Peptides, and Recombinant Proteins**		

Phosphate buffered saline tablets	Sigma-Aldrich	Cat. No. P4417
Biotinylation kit	Avidity, LLC	N/A
Sensor Chip Protein A	Cytiva	Cat#29127555
Dulbecco’s Modified Eagle Medium, high glucose	Sigma-Aldrich	Cat# D5796
Fetal Bovine Serum	GIBCO	Cat# 12676029
Polyethylenimine, branched	Sigma-Aldrich	Cat# 408727
Anti-SARS-Cov IgG CR3022 Kappa chain VJ	ter Meulen et al., 2006	GenBank: ABA54614.1
Anti-SARS-Cov IgG CR3022 Heavy chain VDJ	ter Meulen et al., 2006	GenBank: ABA54613.1

**Deposited Data**		

Crystal structure of SARS-CoV-2 RBD/CR3022 Fab complex (crystal form 1)	This paper	PDB: 6YM0
Crystal structure of RBD/CR3022 Fab complex (crystal form 2)	This paper	PDB: 6YLA
EM structure of CR3022 Fab bound SARS-CoV-2 spike glycoprotein dimer	This paper	EMDB: EMD-10863; PDB: 6YOR
EM structure of SARS-CoV-2 spike glycoprotein	This paper	EMDB: EMD-11119; PDB: 6Z97

**Experimental Models: Cell Lines**		

HEK293S GnTI- cells	ATCC	Cat# CRL-3022
HEK293 cells	TATCC	Cat# CRL-3216
Expi293F™ Cells	GIBCO	Cat# A14527
Vero E6 cells	ECACC, PHE, UK	Cat# 85020206
Hamster ExpiCHO cells	Thermo Fisher	Cat# A29133

**Recombinant DNA**		

Vector: pHLsec	Aricescu et al., 2006	N/A
Vector: pOPING-ET	Nettleship et al., 2008	N/A
Human ACE2 cDNA	Sourcebiosciences	ID: 5297380
Cloning vector AbVec-hIgG1	PMID: 20935207	GenBank: FJ475055
Cloning vector AbVec-hIgKappa	PMID: 20935207	GenBank: FJ475056

**Software and Algorithms**		

Xia2-dials	Winter et al., 2018	https://xia2.github.io/parameters.html
PHENIX	Liebschner et al., 2019	https://www.phenix-online.org/
COOT	Emsley and Cowtan, 2004	https://www2.mrc-lmb.cam.ac.uk/personal/pemsley/coot/
Data Acquisition Software 11.1.0.11	Fortebio	https://www.fortebio.com/products/octet-systems-software
Data Analysis Software HT 11.1.0.25	Fortebio	https://www.fortebio.com/products/octet-systems-software
CryoSPARC v2.15.1-live	Structura Biotechnology Inc.	https://cryosparc.com/
EPU	Thermo Fisher	https://www.thermofisher.com/uk/en/home/electron-microscopy/products/software-em-3d-vis/epu-software.html
Vagabond	HMG unpublished	https://github.com/helenginn/vagabond
Prism 8.0	GraphPad	https://www.graphpad.com/scientific-software/prism/
Biacore T200 Evaluation Software 3.1	Cytiva	www.cytivalifesciences.com

**Other**		

X-ray data were collected at beamline I03, Diamond Light Source, under proposal mx19946 for COVID-19 rapid access	This paper	https://www.diamond.ac.uk/covid-19/for-scientists/rapid-access.html
Cryo-EM data were collected at eBIC, Diamond, under proposal BI26983-2 for COVID-19 rapid access	This paper	https://www.diamond.ac.uk/covid-19/for-scientists/rapid-access.html
TALON® Superflow Metal Affinity Resin	Clontech	Cat. No. 635668
HiLoad® 16/600 Superdex® 200 pg	Cytiva	Cat. No. 28-9893-35
Amine Reactive Second-Generation (AR2G) Biosensors	Fortebio	Cat# 18-5092
Octet RED96e	Fortebio	https://www.fortebio.com/products/label-free-bli-detection/8-channel-octet-systems
QuixStand	GE Healthcare	Cat# 56-4107-78

### Resource Availability

#### Lead Contact

Further information and requests for resources and reagents should be directed to and will be fulfilled by the Lead Contact, David I Stuart (dave@strubi.ox.ac.uk).

#### Materials Availability

Plasmids generated in this study are available from the Lead Contact with a completed Materials Transfer Agreement.

#### Data and Code Availability

The high resolution and lower resolution coordinates and structure factors of the SARS-CoV-2 RBD/CR3022 complex are available from the PDB with accession codes PDB:6YLA andPDB:6YM0 respectively (https://www.rcsb.org/). EM maps and structure models are deposited in EMDB and PDB with accession codes EMDB:EMD-11119 andPDB:6Z97 for the prefusion spike, and EMDB:EMD-10863 and PDB:6YOR for the dimeric RBD/CR3022 complex respectively (https://www.emdataresource.org/). The data that support the findings of this study are available from the corresponding authors on request.

### Experimental Model and Subject Details

#### Bacterial Strains and Cell Culture

DH5α bacteria (Thermo Fisher Cat# 18263012) growing in LB media (Sigma cat# L3397) at 37°C were used for cloning and amplification of plasmid DNA for mammalian cell transfection. Mammalian cells HEK293S GnTI- (ATCC® CRL-3022) or HEK293T (ATCC® CRL-3216) were grown in DMEM media supplemented with 10% Fetal Bovine Serum (FBS, GIBCO, Cat# 12676029) at 30 or 37°C with 5 or 8% CO_2_ respectively. For large scale production of spike ectodomain the same type of cells were grown in roller bottles (Greiner, cat# 681075) without CO_2,_ in DMEM media with 2% FBS at 30°C. Transient expression of RBD, ACE2, CR3022 Fab and CR3022 IgG used Expi293F cells (Thermo Fisher, Cat# A14527) grown in Expi293 Expression Medium (Thermo Fisher Cat# A1435103) in suspension with 8% CO_2_ at 30 or 37°C and shaking at 130 rpm. Vero E6 cells (ECACC 85020206; PHE, UK) were cultured in maintenance medium (minimum essential medium (GIBCO, Cat# 21090-022) with 2 mM L-glutamine (GIBCO, Cat# A2916801), 1% non-essential amino acids (GIBCO, Cat# 11140035), 25 mM HEPES buffer (GIBCO, Cat# 15630056) and 10% heat-inactivated (56°C for 30 min) fetal bovine serum (Sigma, Cat# F4135-500ML) at 37°C for PRNTs. ExpiCHO-S cells and ExpiCHO expression medium (Thermo Fisher, Cat# A14527) were used at 37°C with 8% CO_2_ for the production of CR3022 IgG for these neutralization experiments.

### Method Details

#### Cloning

**CR3022:** Two vectors were constructed containing resident human Cκ and IgG1 CH1 sequences and a signal sequence. Synthetic genes encoding the constant regions were inserted by Infusion® cloning into PmeI-HindIII cut pOPING-ET (Nettleship et al., 2008). The vectors have been engineered so that VL and VH sequences can be inserted into the KpnI- BsiWI (pOPINhuVL) and KpnI-SfoI (pOPINhuVH) restriction sites by Infusion® cloning. Synthetic genes encoding the candidate variable regions of CR3022 (ter Meulen et al., 2006) were purchased from IDT Technologies (Leuven, Belgium) as gBlocks. The VH gene was amplified using the forward primer:5′- GGTTGCGTAGCTGGTACCCAGATGCAGCTGGTGCAATC-3′ and the reverse primer: 5′- GCCCTTGGTGGAGGCGACGGTGACCGTGGTCCCTTG; the VL gene was amplified using the forward primer 5′- GGTTGCGTAGCTGGTACCGACATCCAGTTGACCCAGTC-3′ and the reverse primer 5′-GTGCAGCCACCGTACGTTTGATTTCCACCTTGGTCCC-3′. The genes were inserted into the pOPIN expression vectors by Infusion® cloning.

The CR3022 hIgG1 heavy chain gene was amplified through joining three fragments (using the forward primer 5′- GCGTAGCTGAAACCGGCCAGATGCAGCTGGTGCAATC-3′ and the reverse primer 5′- GCCCTTGGTGGAGGCGCTAGAGACGGTGACCGTGGTCCCTTG-3′, and the CR3022 VH as template; the forward primer 5′- CAAGGGACCACGGTCACCGTCTCTAGCGCCTCCACCAAGGGC-3′ and the reverse primer 5′- CGGTGGGCATGTGTGAGTTTTGTCACAAGATTTGGGCTCAAC-3′, and the CR3022 VH as template; the forward primer 5′- GTTGAGCCCAAATCTTGTGACAAAACTCACACATGCCCACCG-3′ and the reverse primer 5′-GTGATGGTGATGTTTACCCGGAGACAGGGAGAGGCTCTTCTG-3′, and the pOPINTTGneoFc as template) using the forward primer 5′-GCGTAGCTGAAACCGGCCAGATGCAGCTGGTGCAATC-3′ and the reverse primer 5′- GTGATGGTGATGTTTACCCGGAGACAGGGAGAGGCTCTTCTG-3′. The gene was inserted into the vector pOPINTTGneo (Nettleship et al., 2015) incorporating a C-terminal His6 tag.

**CR3022 used for neutralization**: The heavy and kappa light variable genes of the antibody were sourced from the GenBank ABA54613.1 and ABA54614.1 respectively and the codon optimized sequences were synthesized by GeneArt. These sequences were cloned into antibody expression vectors (GenBank FJ475055 and FJ475056). Antibody was expressed using ExpiCHO expression system according to the manufacturer’s protocol and purified using a Protein A MabSelect SuRE column (GE Healthcare). The wash buffer contained 20mM Tris & 150mM NaCl buffered to pH 8.6 and the elution was done using 0.1 M citric acid pH 2.5. The eluate was neutralized immediately using 1.5 M Tris pH 8.6 and then buffer exchanged to PBS using a 15 mL 30 kDa MWCO centrifugal filter (Merck Millipore).

**RBD:** The gene encoding amino acids 330-532 of the Receptor Binding Domain (RBD) of SARS-CoV-2 (Gene ID: MN908947) was amplified from a synthetic gene (IDT Technologies) using the forward primer 5′- GCGTAGCTGAAACCGGCCCGAATATCACAAATCTTTGTCC-3′ and the reverse primer 5′- GTGATGGTGATGTTTATTTGTACTTTTTTTCGGTCCGC-3′ or the reverse primer 5′- GTGATGGTGATGTTTTTCATGCCATTCAATCTTTTGTGCCTCAA AAATATCATTCAAATTTGTACTTTTTTTCGGTCCGC-3′ and inserted into the vector pOPINTTGneo incorporating either a C-terminal His6 or BirA-His6 tag.

**ACE2:** The gene encoding amino acids 19-615 of the human ACE2 was amplified from a an image clone (Sourcebiosciences, clone ID: 5297380) using the forward primer 5′- GCGTAGCTGAAACCGGCTCCACCATTGAGGAACAGGCC-3′ and the reverse primer 5′- GTGATGGTGATGTTTGTCTGCATATGGACTCCAGTC-3′ and inserted into the vector the vector pOPINTTGneo incorporating a C-terminal His6. The gene was also amplified using the forward primer 5′- GCGTAGCTGAAACCGGCTCCACCATTGAGGAACAGGCC-3′ and the reverse primer 5′- CAGAACTTCCAGTTTGTCTGCATATGGACTCCAGTC-3′ and inserted into the vector pOPINTTGneoFc incorporating a C-terminal hIgG1Fc-His6 tag.

**Spike ectodomain:** The gene encoding amino acids 1-1208 of the SARS-CoV-2 spike glycoprotein ectodomain, with mutations of RRAR > GSAS at residues 682-685 (the furin cleavage site) and KV > PP at residues 986-987, as well as inclusion of a T4 fibritin trimerisation domain, a HRV 3C cleavage site, a His-8 tag and a Twin-Strep-tag at the C terminus. As reported by Wrapp et al. (Wrapp et al., 2020)

**Validation and protein production:** All vectors were sequenced to confirm clones were correct. Recombinant RBD, ACE2, CR3022 Fab and CR3022 IgG were transiently expressed in Expi293 and proteins were purified from culture supernatants by an immobilised metal affinity using an automated protocol implemented on an ÄKTAxpress (GE Healthcare, UK) (Nettleship et al., 2009), followed by a Hiload 16/600 superdex 75 or a Superdex 200 10/300GL column, using phosphate-buffered saline (PBS) pH 7.4 buffer. Recombinant spike ectodomain was expressed by transient transfection in HEK293S GnTI- cells for 9 days (Aricescu et al., 2006). Conditioned media was dialysed against 2x phosphate buffered saline pH 7.4 buffer. The spike ectodomain was purified by immobilised metal affinity chromatography using Talon resin (Takara Bio) charged with cobalt followed by size exclusion chromatography using HiLoad 16/600 Superdex 200 column in 150 mM NaCl, 10 mM HEPES pH 8.0, 0.02% NaN_3_ at 4°C, before buffer exchange into 2 mM Tris pH 8.0, 200 mM NaCl (Wrapp et al., 2020).

#### Surface Plasmon Resonance

Surface plasmon resonance experiments were performed using a Biacore T200 (GE Healthcare). All assays were performed with a running buffer of PBS pH 7.4 supplemented with 0.005% v/v Surfactant P20 (GE Healthcare) at 25°C. To determine the binding kinetics between the RBD of SARS-CoV-2 and CR3022 mAb, two different experimental settings were attempted. The first experiment was performed with the use of a CAP sensor chip (GE Healthcare). Biotin CAPture Reagent provided in the Biotin CAPture Kit (GE Healthcare) was captured onto the sensor chip according to manufacturer’s instructions. The RBD with a BirA tag was biotinylated using a biotinylation kit (Avidity, LLC) and was immobilized through the Biotin CAPture Reagent, at a density of 15-30 RU on the sample flow cell. The reference flow cell was left blank. The CR3022 Fab was injected over the two flow cells at a range of five concentrations prepared by serial two-fold dilution from 95 nM, at a flow rate of 30 μL/min using a Single-cycle kinetics program with an association time of 60 s and a dissociation time of 60 s. Running buffer was also injected using the same program for background subtraction. The second experiment was performed using a Sensor Chip Protein A (GE Healthcare). CR3022 IgG was immobilised at a density of approximately 30 RU on the sample flow cell. The reference flow cell was left blank. The RBD was injected over the two flow cells at a range of five concentrations prepared by serial two-fold dilution from 100 nM, at a flow rate of 30 μL/min using a Single-cycle kinetics program with an association time of 75 s and a dissociation time of 60 s. Running buffer was also injected using the same program for background subtraction. All data were fitted to a 1:1 binding model using the Biacore T200 Evaluation Software 3.1. In the competition assay where CR3022 IgG was used as the ligand, approximately 1000 RU of CR3022 IgG was immobilised onto a Sensor Chip Protein A. The following samples were injected: (1) 1 μM ACE2, (2) 1 μM (non-binding anti-Caspr2 control) E08R Fab; (3) a mixture of 1 μM ACE2 and 0.1 μM RBD, (4) a mixture of 1 μM E08R Fab and 0.1 μM RBD, and (4) 0.1 μM RBD. In the competition assay where ACE2-hIgG1Fc was used as the ligand, approximately 1000 RU of ACE2-hIgG1Fc was immobilised onto a Sensor Chip Protein A. The following samples were injected: (1) 1 μM CR3022 Fab, (2) 1 μM E08R Fab; (3) a mixture of 1 μM CR3022 Fab and 0.1 μM RBD, (4) a mixture of 1 μM E08R Fab and 0.1 μM RBD, and (4) 0.1 μM RBD. All injections were performed with an association time of 60 s and a dissociation time of 600 s. All curves were plotted using GraphPad Prism 8 (www.graphpad.com).

#### Bio-layer Interferometry

To further validate the SPR results the K_D_ of Fab CR3022 for RBD was also measured by bio-layer interferometry. Kinetic assays were performed on an Octet Red 96e (ForteBio) at 30°C with a shake speed of 1000 rpm. Fab CR3022 was immobilized onto amine reactive 2nd generation (AR2G) biosensors (ForteBio) and serially diluted RBD (80,40,20,10 and 5 nM) was used as analyte. PBS (pH 7.4) was used as the assay buffer. Recorded data were analyzed using the Data Analysis Software HT v11.1 (Fortebio), with a global 1:1 fitting model.

#### Neutralisation

A preparation of CR3022 (pH 7.2), at a starting concentration of 1.36 mg/mL, was diluted 1 in 160, then dilutions were made 2-fold up to 327,680. SARS-CoV-2 (Australia/VIC01/2020) (Caly et al., 2020) was diluted to a concentration of 933 pfu ml^-1^ (70 pfu/75 μl) and mixed 50:50 in minimal essential media (MEM) (Life Technologies, California, USA) containing 1% (v/v) fetal calf serum (FCS) (Life Technologies) and 25 mM HEPES buffer (Sigma) with doubling antibody dilutions in a 96-well V-bottomed plate. The plate was incubated at 37°C in a humidified box for 1 h to allow neutralization to take place, before the virus-antibody mixture was transferred into the wells of a twice DPBS-washed 24-well plate containing confluent monolayers of Vero E6 cells. Virus was allowed to adsorb onto cells at 37°C for a further h in a humidified box before being overlaid with MEM containing 1.5% carboxymethylcellulose (Sigma, Dorset, UK), 4% (v/v) FCS and 25mM HEPES buffer. After 5 days incubation at 37°C in a humidified box, the plates were fixed overnight with 20% formalin/PBS (v/v), washed with tap water and then stained with 0.2% crystal violet solution (Sigma) and plaques were counted. A mid-point probit analysis (written in R programming language for statistical computing and graphics) was used to determine the dilution of antibody required to reduce SARS-CoV-2 viral plaques by 50% (PRNT50) compared with the virus only control (n = 5). The script used in R was based on a source script from (Johnson et al., 2013). Antibody dilutions were run in duplicate and an internal positive control for the PRNT assay was also run in duplicate using a sample of heat-inactivated (56°C for 30 min) human MERS convalescent serum known to neutralize SARS-CoV-2 (National Institute for Biological Standards and Control, UK). This protocol was repeated in two further experiments with CR3022 (from a different batch) at a starting concentration of 1 mg/mL to compare leaving the virus/antibody mixture on the plate and in parallel with washing it off before the addition of overlay media.

#### Crystallization, Data Collection and X-ray Structure Determination

Purified and deglycosylated RBD and CR3022 Fab were concentrated to 8.3 mg/mL and 11 mg/mL respectively, and then mixed in an approximate molar ratio of 1:1. Crystallization screen experiments were carried out using the nanolitre sitting-drop vapor diffusion method in 96-well plates as previously described (Walter et al., 2003, 2005). Crystals were initially obtained from Hampton Research PEGRx HT screen, condition 63 containing 0.1 M sodium malonate, 0.1 M Tris pH 8.0 and 30% w/v polyethylene glycol 1,000. The best crystals were grown in drops containing 200 nL sample and 100 nL reservoir solution.

Crystals were mounted in loops and frozen in liquid nitrogen prior to data collection. Diffraction data were collected at 100 K at beamline I03 of Diamond Light Source, UK. Diffraction images of 0.1° rotation were recorded on an Eiger2 XE 16M detector (exposure time of either 0.002 s or 0.01 s per frame, beam size 80 × 20 μm and 100% beam transmission). Data were indexed, integrated and scaled with the automated data processing program Xia2-dials (Winter, 2010; Winter et al., 2018). The dataset of 720° was collected from a single frozen crystal to 4.4 Å resolution with 52-fold redundancy. The crystal belongs to space group *P4*_*1*_*2*_*1*_*2* with unit cell dimensions *a* = *b* = 150.5 Å and *c* = 241.6 Å. The structure was determined by molecular replacement with PHASER (McCoy et al., 2007) using search models of human germline antibody Fabs 5-51/O12 (PDB ID, 4KMT (Teplyakov et al., 2014)) heavy chain and IGHV3-23/IGK4-1 (PDB ID, 5I1D (Teplyakov et al., 2016)) light chain, and RBD of SARS-CoV-2 RBD/ACE2 complex (PDB ID, 6M0J (Lan et al., 2020)). There is one RBD/CR3022 complex in the crystal asymmetric unit, resulting in a crystal solvent content of ~87%.

During optimization of the crystallization conditions, a second crystal form was found to grow in the same condition with similar morphology. A dataset of 720° rotation with data extending to 2.4 Å was collected on beamline I03 of Diamond from one of these crystals (exposure time 0.004 s per 0.1° frame, beam size 80 × 20 μm and 100% beam transmission). The crystal also belongs to space group *P4*_*1*_*2*_*1*_*2* but with significantly different unit cell dimensions (*a* = *b* = 163.1 Å and *c* = 189.1 Å). There were two RBD/CR3022 complexes in the asymmetric unit and a solvent content of ~74%.

#### X-ray Crystallographic Refinement and Electron Density Map Generation

The initial structure was determined using the lower resolution data from the first crystal form. Data were excluded at a resolution below 35 Å as these fell under the beamstop shadow. One cycle of REFMAC5 (Murshudov et al., 2011) was used to refine atomic coordinates after manual correction in COOT (Emsley and Cowtan, 2004) to the protein sequence from the search model. The software suite Vagabond was used to convert the atomic model into a bond-based description suited for low resolution refinement (Ginn, submitted). This described the protein model through a series of identical but positionally displaced conformers (referred to as an ensemble). The flexibility was described through whole-molecule translations and rotations per polypeptide chain and intramolecular flexibility through variation in torsion angles of bonds connecting C-alpha atoms. These torsion variations were constrained, with bonds of a similar effect on the flexibility of the protein structure moving in tandem. A global B factor of 130 was applied to the model to account for most of the disorder in the crystal. Alternate rounds of refinement were performed of (a) these flexibility parameters and (b) rigid body refinement of each polypeptide chain, for both the target function was the correlation coefficient with the electron density in real space. Local adjustments of atoms were performed in COOT (Emsley and Cowtan, 2004) using the Vagabond map and average model output coordinates. After local real-space refinement, updated coordinates were reloaded into Vagabond and bond torsion angles were adjusted to match them. Best electron density maps accounting for sources of phase error were output as a list of Fourier coefficients. Maps were sharpened by applying a B factor of −100 (Figure S6). The final refined structure had an R_work_ of 0.331 (R_free_, 0.315) for all data to 4.36 Å resolution. This structure was later used to determine the structure of the second crystal form, which has been refined with PHENIX (Liebschner et al., 2019) to R_work_ = 0.213 and R_free_ = 0.239 for all data to 2.42 Å resolution. This refined model revealed the presence of one extra residue at each heavy chain N terminus and 3 extra residues at the N terminus of one RBD from the signal peptide. There is well ordered density for a single glycan at each of the glycosylation sites at N331 and N343 in one RBD, and only one at N343 in the second RBD.

Data collection and structure refinement statistics are given in Table S3. Structural comparisons used SHP (Stuart et al., 1979), residues forming the RBD/Fab interface were identified with PISA (Krissinel and Henrick, 2007), figures were prepared with PyMOL (The PyMOL Molecular Graphics System, Version 1.2r3pre, Schrödinger, LLC).

#### CR3022 Fab Complex Preparation and cryo-EM Data Collection

Purified spike protein was buffer exchanged into 2 mM Tris pH 8.0, 200 mM NaCl, 0.02% NaN_3_ buffer using a desalting column (Zeba, Thermo Fisher). A final concentration of 0.2 mg/mL was incubated with CR3022 Fab (in the same buffer) in a 6:1 molar ratio (Fab to trimeric spike) at room temperature. Aliquots were taken at 50 min and 3 h and 3 μL immediately applied to a holey carbon-coated 200 mesh copper grid (C-Flat, CF-2/1, Protochips) that had been freshly glow discharged on high for 20 s (Plasma Cleaner PDC-002-CE, Harrick Plasma) and excess liquid removed by blotting for 6 s with a blotting force of −1 using vitrobot filter paper (grade 595, Ted Pella Inc.) at 4.5°C, 100% relative humidity. Blotted grids were then immediately plunge frozen using a Vitrobot Mark IV (Thermo Fisher).

Frozen grids were first screened on a Glacios microscope operating at 200 kV (Thermo Fisher) before imaging on a Titan Krios G2 (Thermo Fisher) at 300 kV. Movies (40 frames each) were collected in compressed tiff format on a K3 detector (Gatan) in super resolution counting mode using a custom EPU version 2.5 (Thermo Fisher) with a defocus range of 0.8-2.6 μm and at a nominal magnification of x105,000, corresponding to a calibrated pixel size of 0.83 Å/pixel, see Table S4.

#### Cryo-EM Data Processing

For both the 50 min and 3 h incubation datasets, motion correction and alignment of 2x binned super-resolution movies was performed using Relion3.1. CTF-estimation with GCTF (v1.06) (Zhang, 2016) and non-template-driven particle picking was then performed within cryoSPARC v2.14.1-live (https://cryosparc.com/) followed by multiple rounds of 2D classification (Punjani et al., 2017).

For the 50 min dataset, 2D class averages for particle groups A and B were used separately for template-driven classification before further rounds of 2D and 3D classification with C1 symmetry. Both structures were then sharpened in cryoSPARC. Data processing and refinement statistics are given in Table S4.

An initial model for the spike (group A) was generated using PDB ID, 6VYB (Walls et al., 2020) and rigid body fitted into the final map using COOT (Emsley and Cowtan, 2004). The model was further refined in real space with PHENIX (Liebschner et al., 2019) which resulted in a correlation coefficient of 0.84. Two copies of RBD-CR3022 were fitted into the map for group B in the same manner. Because of the strongly anisotropic resolution the overall correlation coefficient versus the model was lower (0.47).

For the 3 h incubation dataset, particles were extracted with a larger box size (686 pixels as compared to 540 pixels), and, following multiple rounds of 2D classification, 2D class averages from ‘blob-picked’ particles showing signs of complete ‘flower-like’ structures were selected for *ab initio* reconstruction (in some classes, petals from these flower-like particles were missing, Figure S7). For the 3 h data no detailed fitting was attempted.

### Quantification and Statistical Analysis

SPR kinetic data were fitted using Biacore T200 Evaluation software 3.1 (www.cytivalifesciences.com). BLI data were analyzed using data analysis software HT V 11.1 (www.fortebio.com). PRNT neutralization data were subjected to mid-point probit analysis based on a source script from Johnson et al., 2013 and 95% confidence intervals are given (also reported in the figure legends and Method Details).

## Supplemental Information

Document S1. Figures S1–S7 and Tables S1–S4

Document S2. Article plus Supplemental Information
