# Supplementary material for: Neutralization of SARS-CoV-2 by Destruction of the Prefusion Spike
Source: Cell Host Microbe. 2020 Sep 9;28(3):445–454.e6. doi: 10.1016/j.chom.2020.06.010 (PMC7303615; doi:10.1016/j.chom.2020.06.010)
Supplement: Document S1. Figures S1–S7 and Tables S1–S4 [file mmc1.pdf]

Cell Host & Microbe, Volume *28*

Supplemental Information

Neutralization of SARS-CoV-2 by Destruction of the Prefusion Spike

Jiandong Huo, Yuguang Zhao, Jingshan Ren, Daming Zhou, Helen M.E. Duyvesteyn, Helen M. Ginn, Loic Carrique, Tomas Malinauskas, Reinis R. Ruza, Pranav N.M. Shah, Tiong Kit Tan, Pramila Rijal, Naomi Coombes, Kevin R. Bewley, Julia A. Tree, Julika Radecke, Neil G. Paterson, Piyada Supasa, Juthathip Mongkolsapaya, Gavin R. Screaton, Miles Carroll, Alain Townsend, Elizabeth E. Fry, Raymond J. Owens, and David I. Stuart

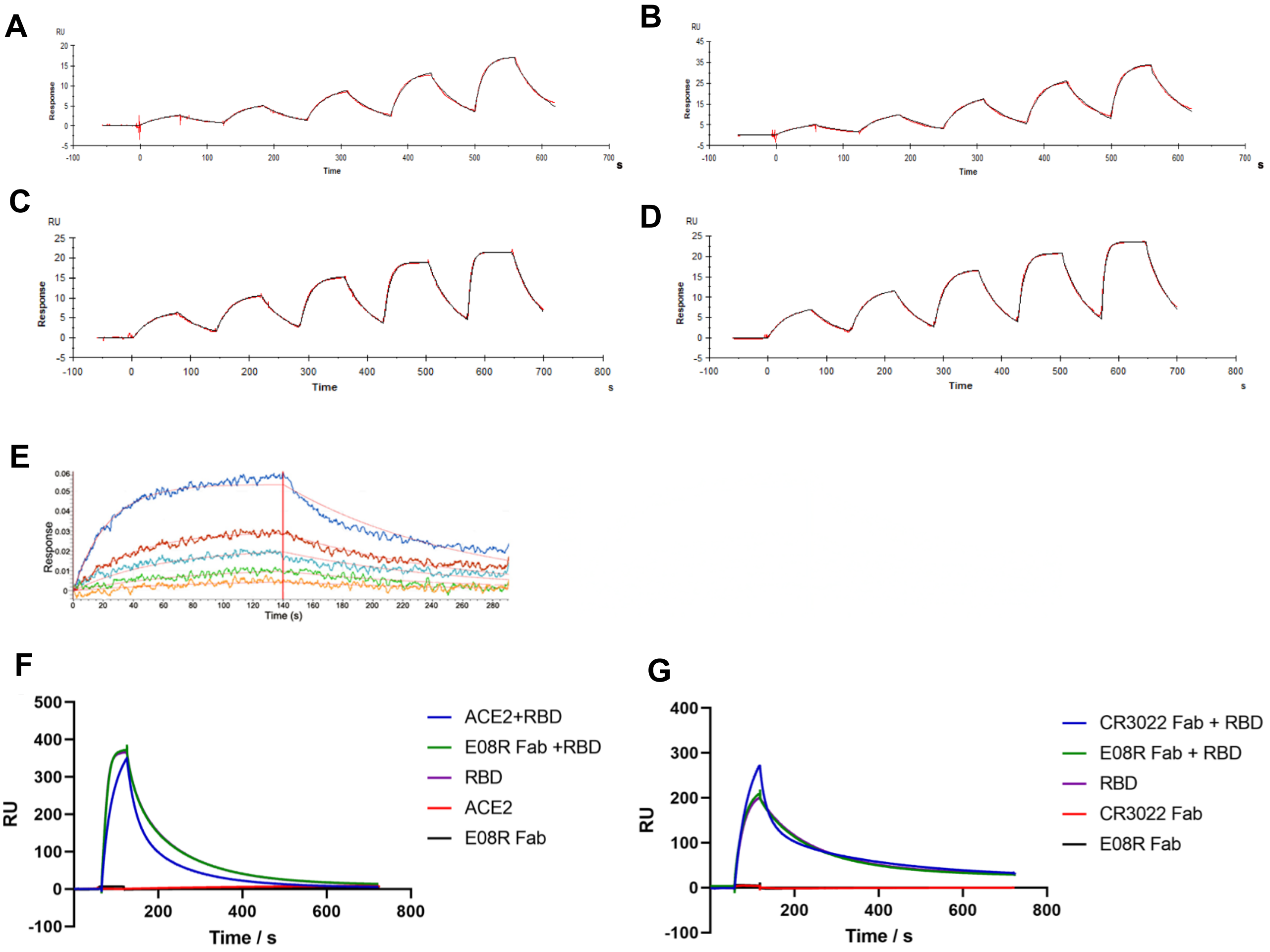

Figure S1 Binding affinity between RBD and CR3022 Fab, related to STAR Methods, (Surface plasmon resonance and Bio-layer Interferometry).

(A-B) Surface plasmon resonance binding sensorgrams measured with a Biacore T200. Biotinylated (Bio-) RBD was immobilised as the ligand and CR3022 Fab was used as analyte at five concentrations (5.9, 11.9, 23.8, 47.5 and 95 nM). (C-D) CR3022 IgG was immobilised as the ligand and RBD-His was used as analyte at five concentrations (6.25, 12.5, 25, 50, 100 nM). Data were fitted to a 1:1 binding model using the Biacore T200 Evaluation Software 3.1. The average kinetic values from these two sets of experiment are listed in Extended Table 1. (E) Binding sensorgram of the interaction between RBD and CR3022 Fab measured with an Octet platform. CR3022 Fab was immobilized onto AR2G biosensors, and RBD was used as analyte with a serial dilution of 5,10, 20, 40 and 80 nM. The measured K_D is 19 nM using a global 1:1 fitting model. Binding competition of ACE2 and CR3022 Fab for RBD: Surface plasmon resonance binding sensorgrams measured with a Biacore T200. (F) CR3022 IgG was immobilised as the ligand, and the following samples were injected: (1) a mixture of 1 μM ACE2 and 0.1 μM RBD; (2) a mixture of 1 μM E08R (a non-binding anti-caspr2 Fab) and 0.1 μM RBD; (3)) 0.1 μM RBD; (4) 1 μM ACE2; (5) E08R Fab. (G) ACE2-hIgG1Fc was immobilised as the ligand, and the following samples were injected: (1) a mixture of 1 μM CR3022 Fab and 0.1 μM RBD; (2) a mixture of 1 μM E08R Fab and 0.1 μM RBD; (3) 0.1 μM RBD; (4) 1 μM CR3022 Fab; (5) 1 μM E08R Fab.

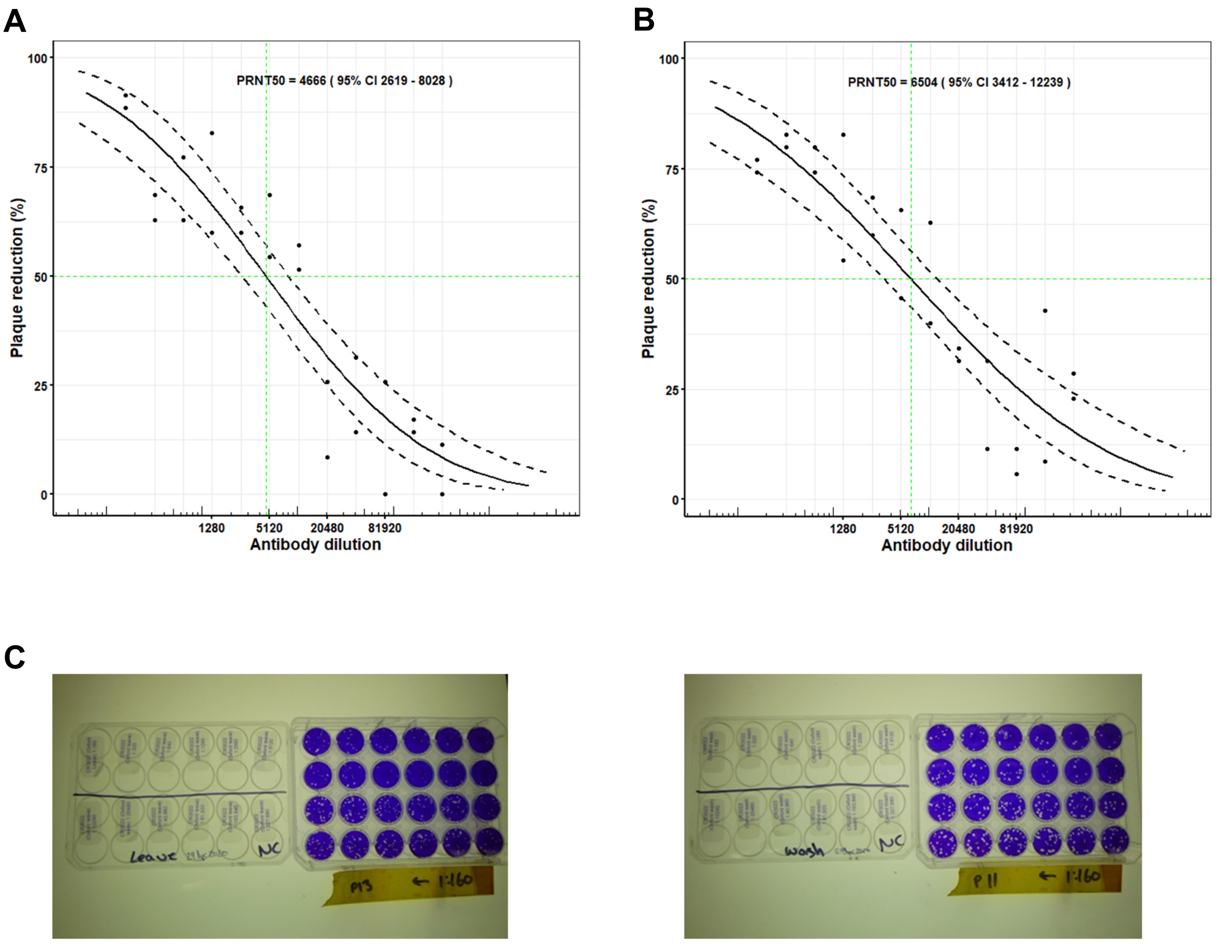

Figure S2 Dose Response Curve for 'leave' and 'wash' PRNT with CR3022, related to Figure 2 and STAR Methods (Neutralisation).
(A) **'**Leave' plate**.** For CR3022 at a starting concentration of 1 mg/mL, the dilutions used were from 1:160 to 1:327,680, the virus/antibody mix was left on the plate. The probit mid-point is 1:4,666 (95% confidence intervals: 2,619-8,028) and the positive control 1:629 (95 % confidence intervals: 382-958). (B) 'Wash' plate. As for (A) but the virus/antibody mix was washed. The probit mid-point is 1:6,504 (95% confidence intervals: 3,412-12,239). The positive control probit mid-point is 1:629 (95 % confidence intervals: 382-958). (C) Plate photographs**.** Note that the plate is photographed transversed relative to the lid, so the 1:160 dilution duplicate wells are in the top right corner, and the 1:327,680 dilution duplicate wells are in the bottom left corner of the stained plate.

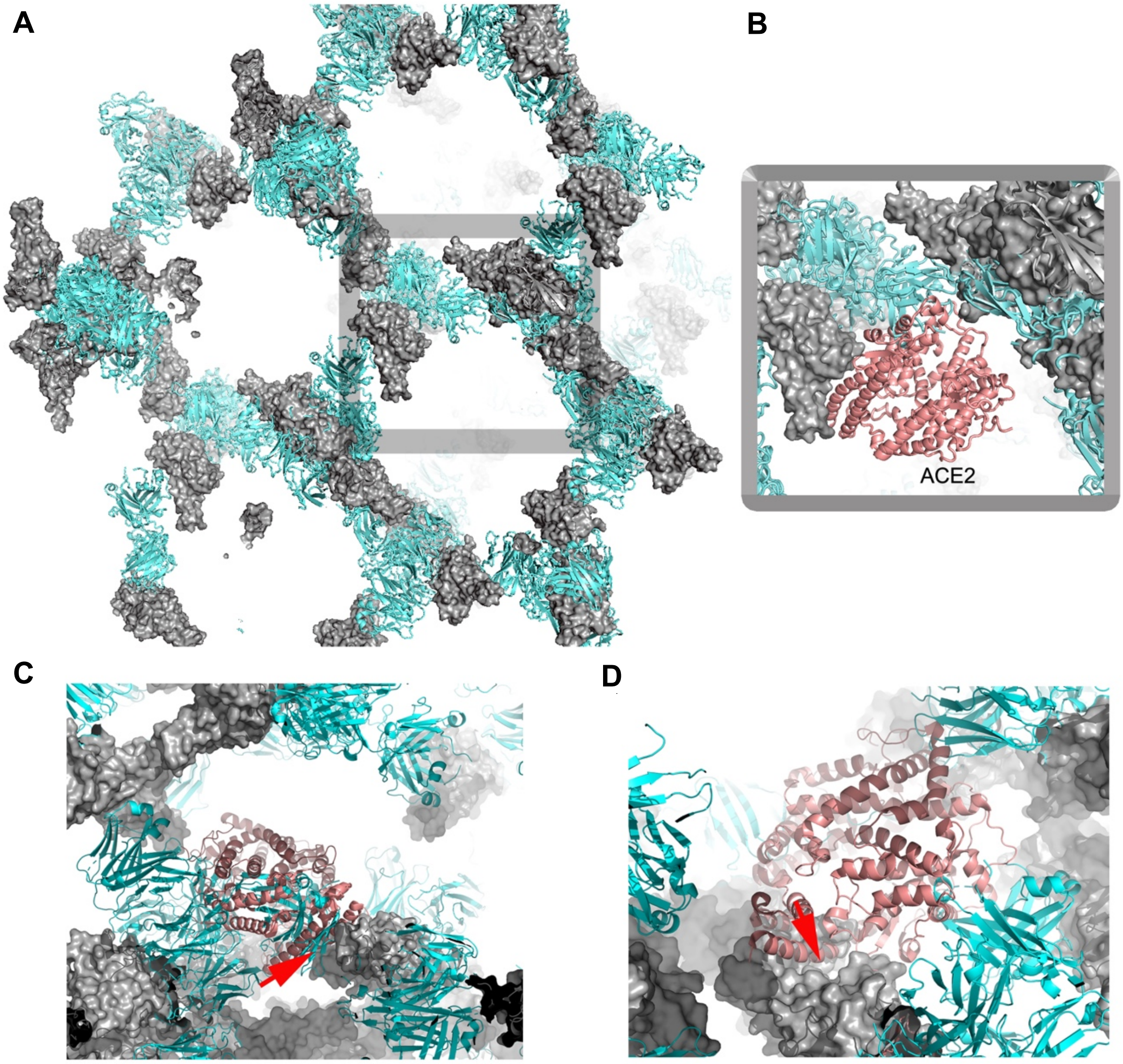

Figure S3 Crystal lattice of the RBD/CR3022 complex, related to Figure 3 and STAR Methods (Crystallisation, data collection and X-ray structure determination).
(A) The packing of the RBD/CR3022 complex within the first crystal form. The RBD is shown as a grey surface and CR3022 Fab as cyan ribbons. (B) A closeup of the crystal lattice with the RBD of the receptor complex overlapped onto the RBD of the Fab complex showing that the receptor binding site of the RBD is not blocked in the crystal. (C-D) The ACE2 binding sites of the 2 RBDs in the second crystal form are blocked by crystal contact (indicated by red arrows).

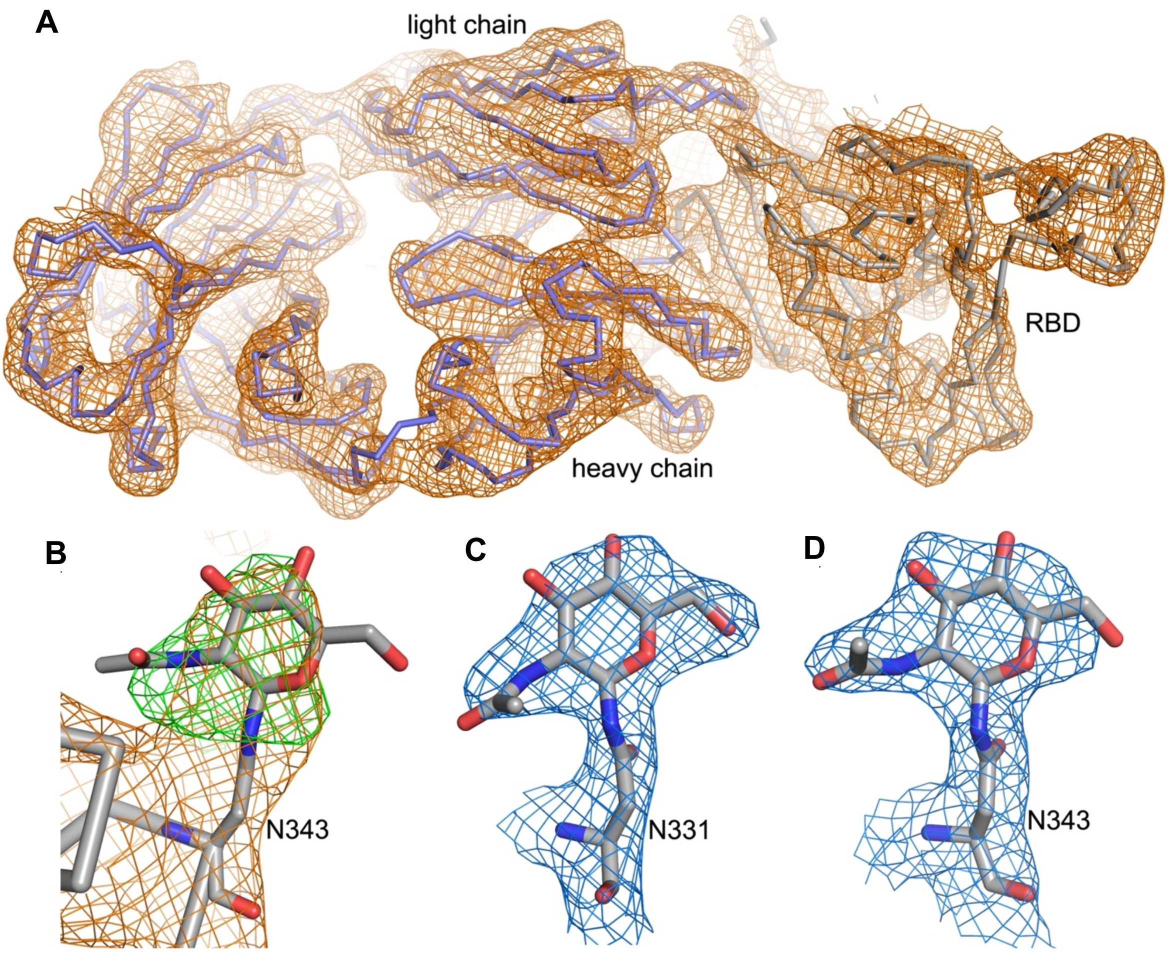

Figure S4 Electron density maps. related to Figure 3 and STAR Methods (X-ray crystallographic refinement and electron density map generation).
(A) 4.4 Å resolution electron density map for crystal form 1, produced with Vagabond (see methods) and contoured at 1.2 σ showing the overall quality of the structure. (B) Difference electron density map (green) contoured at 3 σ showing the glycosylation site at N343 of the RBD. The glycan was not modelled into the structure used for the map calculation. (C-D), Electron density maps of the glycosylation sites N331 (C) and N343 (D) in the second, high resolution (2.4 Å), crystal form.

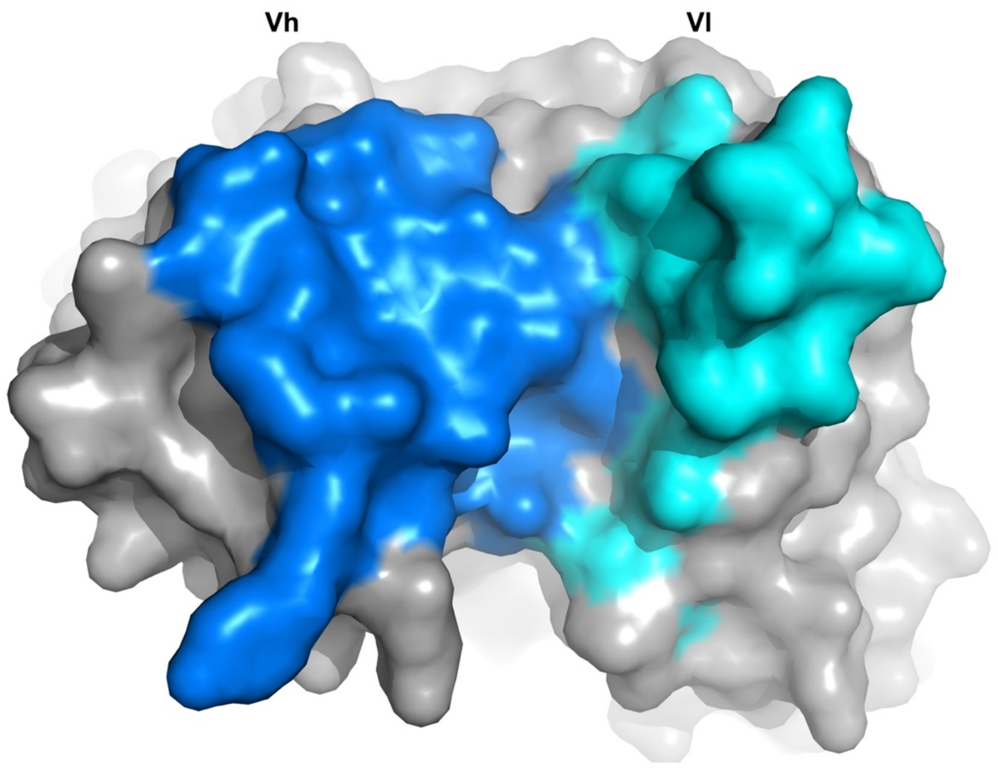

Figure S5 Buried solvent accessible area of the CR3022 antigen binding region due to engagement with the RBD, related to Figure 4.
Buried areas are coloured in blue for the heavy chain (Vh) and cyan for the light chain (Vl).

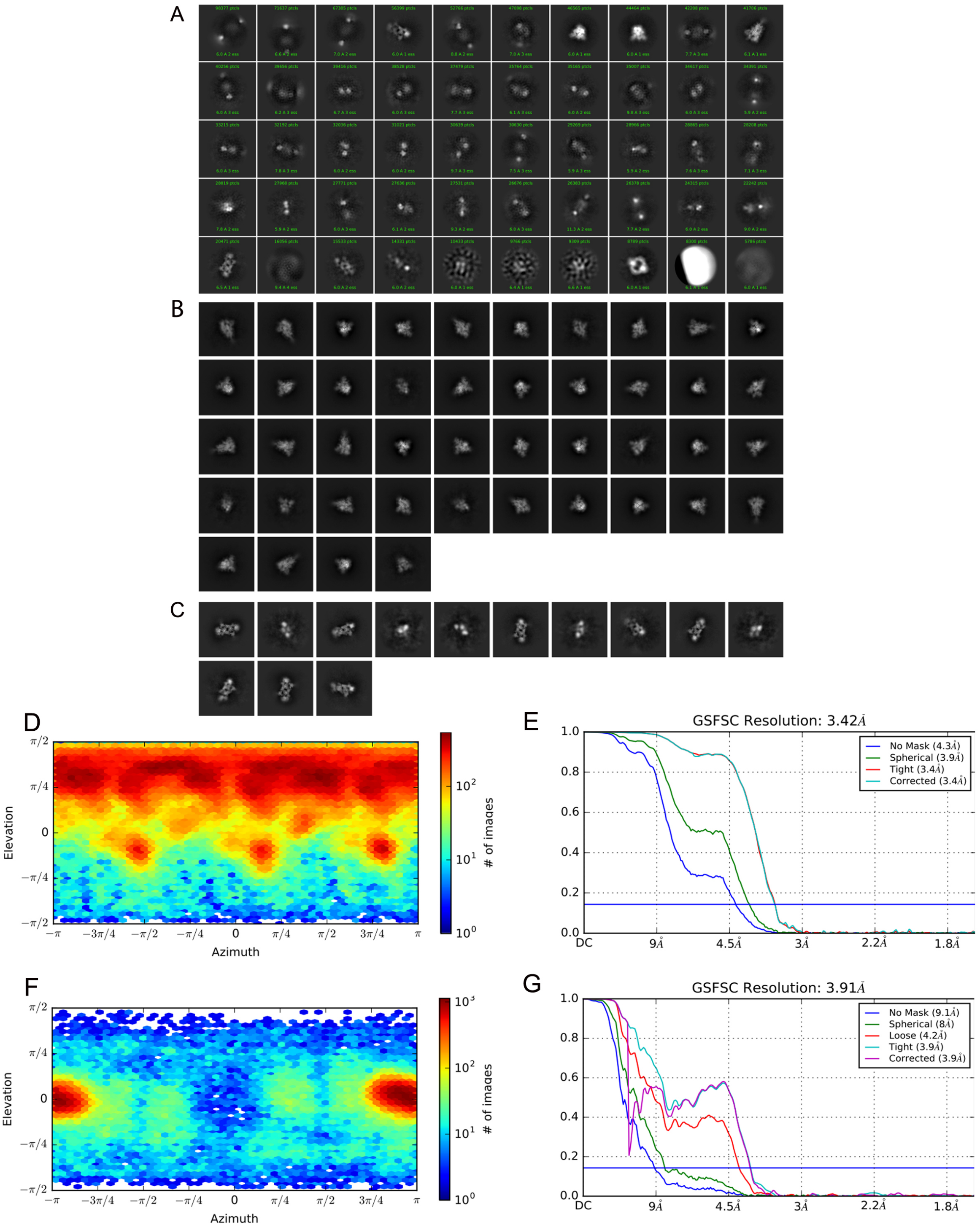

Figure S6 Cryo-EM analysis of 50 minutes incubation, related to Figure 7 and STAR Methods (Cryo-EM data processing).

2D class averages: (A) unbiased classes from blob picked particles, with particle numbers and estimated resolutions in green text (CryoSPARC). (B) Selected classes for the prefusion state reconstruction. (C) Selected classes for the dimeric association reconstruction. Prefusion Spike analysis: (D) orientation distribution, (E) Gold standard FSC analysis from CryoSPARC, with FSC = 0.143 marked with a horizontal solid blue line. Dimeric CR3022/RBD: (F) orientation distribution. (G) Gold standard FSC plot generated in CryoSPARC, with FSC = 0.143 marked with a horizontal blue line. See Methods for details.

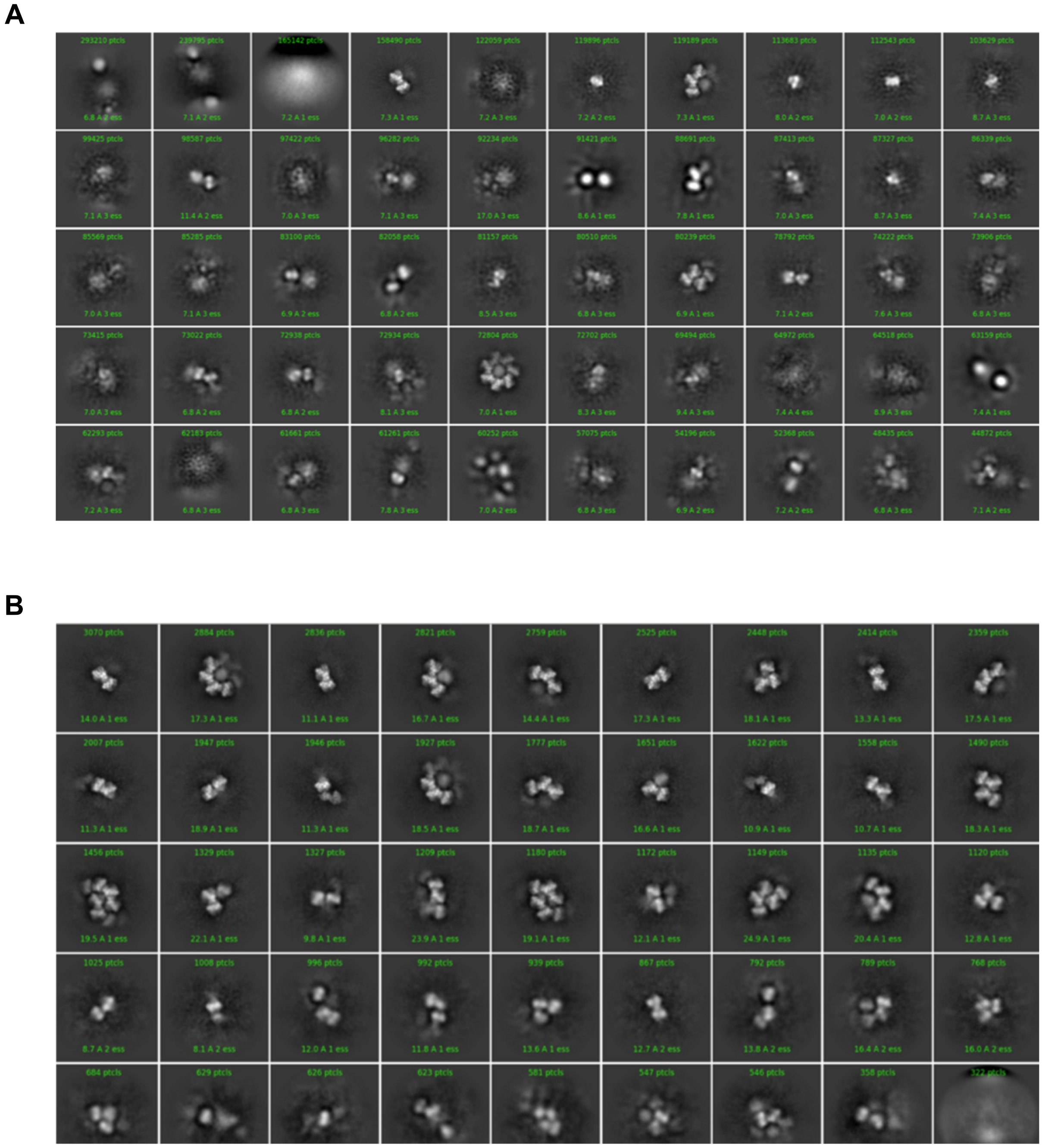

Figure S7 Analysis of cryo-EM data for 3 h incubation, related to Figure 7 and STAR Methods (cryo-EM data processing).
2D class averages: (A) unbiased classes i.e. following a single round of blob-picked particles in cryoSPARC, (B) selected classes showing oligomeric assemblies.

Table S1 SPR kinetic results, related to Figure S1 and STAR Methods (Surface plasmon resonance).

**Ligand**	**Biotinylated RBD**	**CR3022 IgG**
Analyte	CR3022 Fab	His-tagged RBD
Ka ($M^{-1}s^{-1}$)	6.3E+05	1.5E+06
Kd (s^{-1})	1.9E-02	2.3E-02
KD (nM)	30	15

Table S2 Plaque Reduction Neutralization Test results, related to Figure 2 and STAR Methods (Neutralisation).

ID (batch number)	Description	$PRNT_{50}$ [95% CI]
(1) Positive control	MERS convalescent serum	1:874 [663-1,220]
(1) CR3022 1.36 mg/mL	Mab (batch 2)	1:11,966 [5,297-23,038]
(2) Positive control	MERS convalescent serum	1:629 [382-958]
(2) CR3022 1.0 mg/mL	Mab (batch 1) Leave on plate	1:4666 [2,619-8,028]
(3) Positive control	MERS convalescent serum	1:629 [382-958]
(3) CR3022 1.0 mg/mL	Mab (batch 1) Wash off plate	1:6504 [3,412-12,239]

Table S3 X-ray data collection and refinement statistics, related to Figure 3 and STAR Methods (Crystallisation, data collection and X-ray structure determination and X-ray crystallographic refinement and electron density map generation).

**Data collection**		
Data set	Crystal form 1	Crystal form 2
Space group	$P4_12_12$	$P4_12_12$
Cell dimensions (Å)	a=150.5, b=150.5, c=241.6	a=163.1, b=163.1, c=189.1
Resolution (Å)	80.5–4.36 (4.44–4.36)	58.8–2.42 (2.46–2.42)
Unique reflections	18822 (931)	97407 (4803)
R_{merge}	0.683 (---)	0.303 (---)
R_{pim}	0.097 (1.597)	0.034 (1.536)
$CC_{1/2}$	0.952 (0.316)	0.997 (0.451)
$\langle I \rangle / \langle \sigma I \rangle$	4.0 (0.2)	11.6 (0.2)
Completeness (%)	100 (100)	100 (100)
Redundancy	51.6 (54.4)	78.7 (78.8)
**Refinement**		
Resolution (Å)	35.0–4.36	55.3–2.42
No. reflections	17940	94155
R_{work} / R_{free}	0.331/0.315	0.213/0.239
No. atoms	4861	10072
Average B-factors ($Å^2$)	151	89
Parameters		
Positional	4298	N/A
Flexibility	2391	N/A
Total	6689	N/A
R.m.s. deviations		
Bond lengths (Å)	N/A	0.002
Bond angles (°)	N/A	0.5

Numbers in brackets refer to the highest resolution shell of data

Table S4 Cryo-EM data collection parameters, related to Figure 7 and STAR Methods (cryo-EM data processing).

	**3h incubation**	**50 min incubation trimer**	**50 min incubation 'dimer'**
**Data collection and reconstruction**			
Voltage (kV)	300		
Frames	40	40	
Dose rate (e^-/ Å ^{2}/ s)	20.2	20.7	
Total dose (e^-/ Å ^{2})	42	42.0	
Pixel size (Å) (super-resolution)	0.415	0.415	
Defocus (μm)	0.8-2.6	0.8-2.6	
Symmetry	C1	C1	
Movies	7,032	13,307	
Particles	24,303	327,945	100,295
Resolution FSC = 0.143 (Å)		3.3	3.9/7.0
Map sharpening B-factor (Å^2)		-111.1	-92.3
**Model refinement**			
Model-to-map fit, CC_mask		0.84	0.47
R.m.s.d., bonds (Å)		0.006	0.002
R.m.s.d., angles (°)		0.9	0.5
All-atom Clash score		7.6	1.8
Rotamer outliers (%)		3.8	2.0
Ramachandran plot			
Favored (%)		95.4	95.9
Allowed (%)		3.8	3.9
Outliers (%)		0.2	0.2
